# Case Report: Opioid-sparing general anesthesia with bilateral quadratus lumborum block for cesarean delivery in a parturient with spinal deformity

**DOI:** 10.3389/fmed.2026.1839041

**Published:** 2026-08-03

**Authors:** Shuanjun Zhang, Juxian Zhao, Fang Yang, Cuiwen Li, Haixia Wei, Wen Li, Zhonghua Bai, Huiwen Wang

**Affiliations:** 1 Department of High Altitude Medicine, The 940th Hospital of Joint Logistic Support Force of Chinese People's Liberation Army, Lanzhou, China; 2 Department of Anesthesiology, The 940th Hospital of Joint Logistic Support Force of Chinese People's Liberation Army, Lanzhou, China

**Keywords:** bilateral quadratus lumborum block, case report, cesarean delivery, opioid-sparing general anesthesia, spinal deformity

## Abstract

For parturients with contraindications to neuraxial anesthesia, GA remains the primary alternative. However, conventional opioid-based GA may increase the risk of adverse neonatal outcomes. Therefore, exploring opioid-sparing GA combined with bilateral QLB is of considerable clinical value for reducing potential maternal and neonatal risks.In the present case, the patient was a 35-year-old full-term parturient with spinal deformity who declined neuraxial anesthesia, constituting a contraindication to such techniques.During cesarean delivery, we successfully implemented an opioid-sparing GA regimen combined with bilateral QLB. The essential components of GA—hypnosis, analgesia, muscle relaxation, and attenuation of the stress response—were achieved through a multimodal approach. The anesthetic management was carefully designed following a comprehensive preoperative evaluation and a thorough analysis of the challenges associated with conventional opioid-based GA in this patient. Multidisciplinary collaboration and meticulous perioperative planning were emphasized throughout the peripartum period. This anesthetic approach effectively integrated the advantages of regional nerve block and GA. Notably, the neonate exhibited no signs of opioid-related respiratory depression, with no occurrence of low Apgar scores, and no resuscitative interventions were required after delivery. This case highlights a novel opioid-sparing strategy for cesarean delivery under GA.

## Introduction

General anesthesia (GA) is one of the most commonly used anesthetic techniques in clinical practice, in which opioids play a pivotal role as the primary analgesic component. However, in cesarean delivery (CD), the use of opioids poses a substantial risk of neonatal respiratory depression, a concern that warrants particular attention. Cesarean delivery is a common obstetric procedure, primarily indicated for conditions such as dystocia, fetal distress, and compromised maternal health. Global data indicate a marked increase in CD rates in both developing countries and certain developed regions (1).

Anesthetic approaches for CD are broadly categorized into neuraxial anesthesia and GA (2). Currently, neuraxial techniques—including epidural anesthesia, spinal anesthesia, and combined spinal–epidural anesthesia—provide effective anesthesia for CD and are considered the preferred modality. Compared with GA, neuraxial anesthesia is associated with fewer postoperative complications, higher maternal satisfaction, enhanced early postoperative recovery, and improved neonatal Apgar scores (3). Nevertheless, for parturients who refuse neuraxial anesthesia or present with contraindications—such as spinal deformity, a history of spinal surgery, spinal infection, or severe coagulopathy (4)—alternative anesthetic strategies must be considered, with GA remaining the principal option (2). Traditionally, opioids constitute an essential component of GA. Shi X et al. (5) demonstrated that, compared with neuraxial anesthesia, parturients undergoing GA exhibited significantly reduced oxygenation indices, while neonates had lower Apgar scores at 1 and 5 min and a higher likelihood of requiring resuscitation. In addition, GA is associated with increased rates of neonatal intensive care unit (NICU) admission, neonatal respiratory distress syndrome (NRDS), and even higher fetal mortality (6). The transplacental transfer of opioids and their consequent respiratory depressive effects on the fetus were considered the primary contributors to these adverse outcomes (7).

Although remifentanil has the advantages of an ultra-short duration of action and unique metabolism, it may still exert adverse effects on neonates (8). Enten G et al. (9) proposed an opioid-free GA regimen incorporating preoperative administration of non-opioid analgesics for preventive analgesia; however, this approach may be limited by insufficient analgesic efficacy. Therefore, exploring alternative analgesic strategies to replace opioids in GA is of significant clinical importance to reduce maternal and neonatal risks. Identifying effective opioid-sparing or opioid-free anesthetic approaches for parturients requiring GA remains an urgent clinical challenge. With the rapid development of ultrasound-guided nerve block techniques, these techniques are increasingly used as adjuncts to GA or neuraxial anesthesia, offering significant analgesic benefits. Among them, the quadratus lumborum block (QLB) provides effective abdominal analgesia (10, 11), although it is most commonly applied for postoperative pain control. In the present case, we adopted opioid-sparing GA combined with bilateral QLB to minimize neonatal adverse effects. In the present case, we adopted opioid-sparing GA combined with bilateral QLB to minimize neonatal adverse effects. At present, clinical experience with the use of opioid-sparing GA with bilateral QLB for cesarean delivery remains limited, and its safety and efficacy require further validation through well-designed clinical studies.

## Case Report

### Clinical data

The patient was a 35-year-old (height 155 cm, weight 65 kg) primigravida (G1P0), Planned management: A cesarean delivery was scheduled based on the diagnosis of term pregnancy, advanced maternal age, precious fetus, and pregnancy complicated by spina bifida with tethered cord syndrome. Pre-anesthetic evaluation revealed hemoglobin (Hb) of 123 g/L, platelet count of 272 × 10?/L, and normal coagulation function. Electrocardiography demonstrated sinus rhythm with a heart rate of 86 beats/min. Physical examination identified a depressed surgical scar in the lumbar region measuring approximately 3 × 5 cm, with a maximum depth of 2 cm. NPO for 8 h. Airway assessment showed a mouth opening of 3.5 cm, Mallampati class III, thyromental distance of 6.5 cm, normal cervical mobility, and no history of significant snoring. Medical history: The parturient had an expected date of delivery on October 23, 2024. There was no rupture of membranes, and uterine contractions were present and gradually regular. She reported undergoing spinal surgery at the age of 9. Spinal MRI performed 2 years prior revealed incomplete neural tube closure, lumbosacral spina bifida occulta, syringomyelia, and tethered cord syndrome. Based on the comprehensive evaluation, the patient was classified as American Society of Anesthesiologists (ASA) physical status II and was considered to have contraindications to neuraxial anesthesia; therefore, GA was recommended. Antenatal evaluation: Prenatal assessments, including nuchal translucency (NT) screening and detailed anomaly scans, indicated normal fetal development. On admission, ultrasonography demonstrated a cephalic (occiput anterior) presentation without nuchal cord. Fetal heart rate monitoring showed a baseline of 140 beats per minute.

### Diagnosis

Term pregnancy; advanced maternal age; precious fetus; pregnancy complicated by spina bifida with tethered cord syndrome.

### Assessment

Following multidisciplinary consultation (obstetrics, neurology, spine surgery, and anesthesiology), and based on the patient's medical history and available investigations, the patient was classified as American Society of Anesthesiologists (ASA) physical status II. Neuraxial anesthesia was considered technically challenging and associated with a potential risk of neurological injury. A repeat MRI and cesarean delivery under GA were therefore recommended. However, given that the patient was near term with regular uterine contractions and reported no significant symptoms related to spina bifida, and considering the availability of alternative anesthetic options, she elected to defer repeat MRI. Due to concerns regarding puncture difficulty and the risk of neurological complications, the patient declined neuraxial anesthesia. Although the anesthesiologist explained that obstetric-specific GA techniques could mitigate potential neonatal effects, the patient remained concerned that GA is not the routine first-line approach in obstetrics and may carry a higher risk of neonatal respiratory depression. After comprehensive evaluation and discussion of the challenges, a plan was formulated to perform bilateral QLB combined with opioid-sparing GA, with a laryngeal mask airway for airway management. This anesthetic strategy was explained in detail, and written informed consent was obtained from the patient.

### Perioperative course

**Figure 1:**
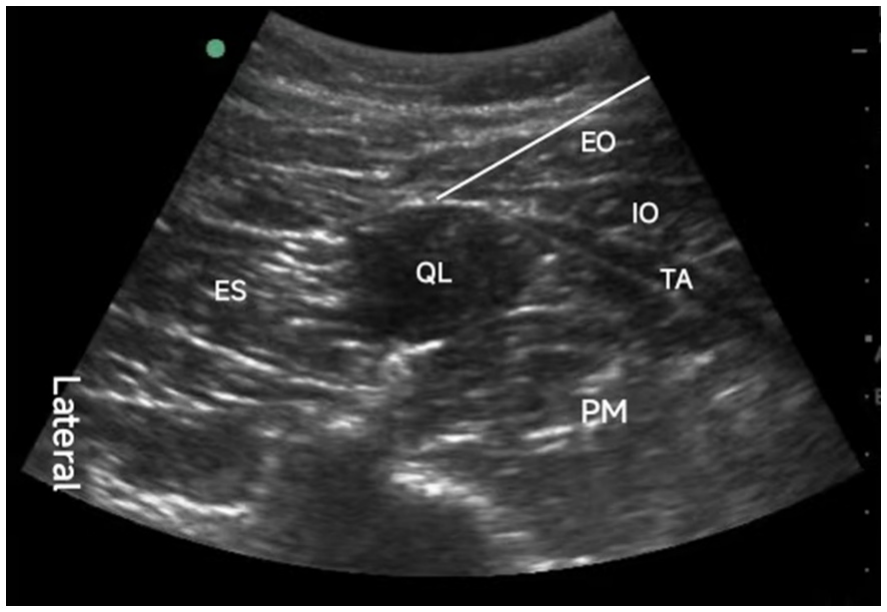
Ultrasound-guided QLB. TP, transverse process; PM, psoas major; QL, quadratus lumborum; ES, erector spinae; EO, External oblique; IO, Internal oblique; TA, Transversus abdominis, White line, needle insertion trajectory.

**Figure 2:**
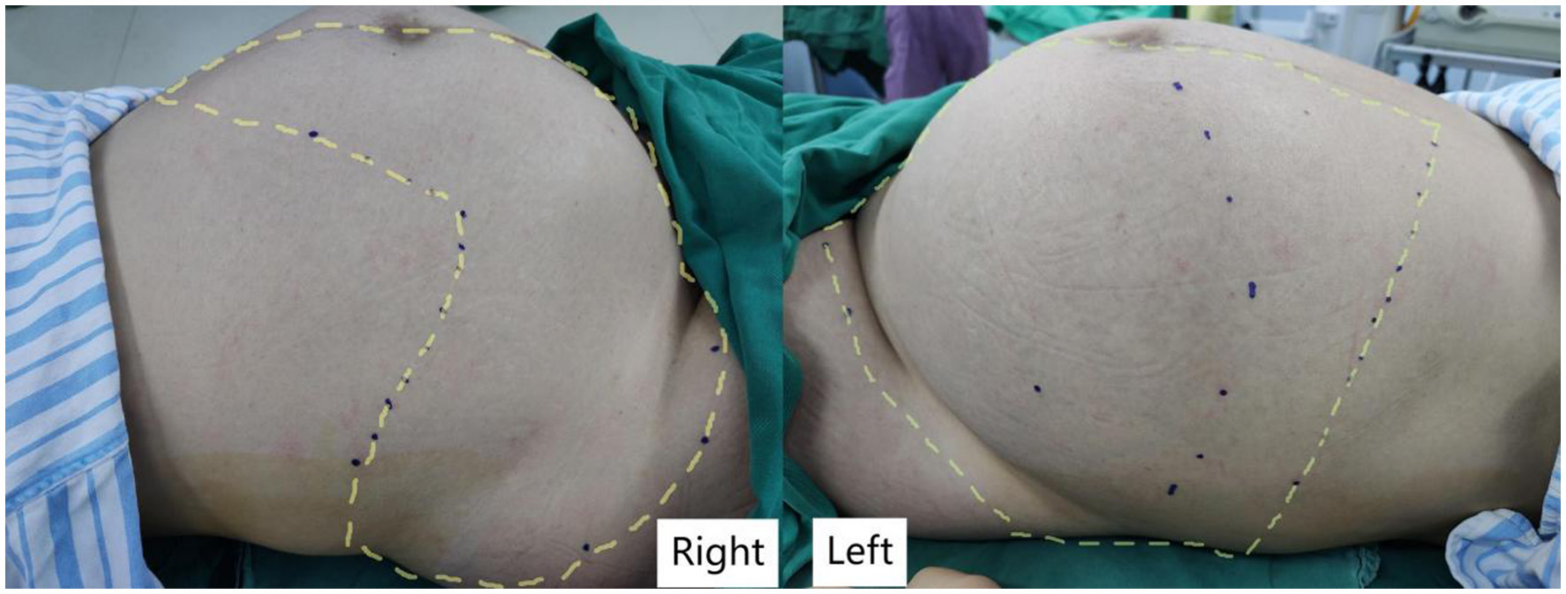
Extent of the sensory blockade following a QLB. The dotted area indicates the region of effective anesthesia, which completely covered the planned surgical incision.

Upon arrival in the operating room, peripheral intravenous access was established in the upper limb. Standard monitoring was applied, including electrocardiography (ECG), pulse oximetry (SpO_2_), noninvasive blood pressure (NIBP), temperature, and Narcotrend monitoring. Preoperative medication consisted of intravenous parecoxib sodium 40 mg. Bilateral QLB was then performed. With the patient in the supine position, a convex array probe (Sonosite M-Turbo) was used, and an in-plane technique was employed to advance the needle to the fascial plane at the lateral border of the quadratus lumborum muscle (Figure 1). A total of 20 mL of a mixture containing 0.5% ropivacaine and dexamethasone 5 mg was injected on each side (12). Ultrasound visualization confirmed appropriate spread of the local anesthetic within the fascial plane. Strict aseptic technique was maintained throughout, and repeated aspiration was performed to avoid intravascular injection. The procedure was repeated contralaterally. The total dose of ropivacaine was 200 mg, and dexamethasone 10 mg. Twenty min later, the sensory block was assessed using an alcohol swab and mapped (Figure 2). The upper level reached the xiphoid process (T6), and the lower level extended to below the inguinal region (L1–L2). The bilateral block distribution was symmetrical and fully covered the surgical incision area, with reduced pinprick sensation confirmed at the incision site. No clinical signs of local anesthetic systemic toxicity were observed.

Rapid sequence induction of GA was then performed. After 5 min of preoxygenation, 150 mg propofol and 60 mg rocuronium were administered intravenously 2 min prior to skin incision. Airway equipment and suction devices were confirmed to be functional, and cricoid pressure was applied to reduce the risk of regurgitation and aspiration. A size 4 LMA Supreme was inserted, and effective ventilation was confirmed before initiating mechanical ventilation. Anesthesia was maintained with TCI propofol infusion (target plasma concentration 2–3 μg/ml) to ensure adequate anesthetic depth. Sevoflurane and remifentanil (20 μg/ml, via TCI or intravenous infusion) were prepared as backup options and could be initiated promptly if required. The cesarean delivery was performed via a suprapubic transverse incision and a lower uterine segment transverse incision. After delivery of the fetus and intact placenta, the abdominal cavity was inspected and cleared, followed by closure of the uterus and abdomen. Intraoperatively, blood pressure was maintained at 120–130/70–80 mmHg, heart rate at 90–100 beats/min, SpO_2_ at 96%−98%, and Narcotrend values ranged between D–E (40–60). During emergence, the patient regained consciousness smoothly. Sugammadex 140 mg was administered intravenously, and the laryngeal mask airway was removed after confirming a train-of-four (TOF) ratio >0.9. Outcomes: A live female neonate weighing 2,560 g was delivered 8 min after the start of surgery, with Apgar scores of 10 at 1, 5, and 10 min. The placenta was delivered intact 5 min later. The mother recovered well, with no immediate anesthetic complications and no intraoperative awareness. Estimated blood loss was approximately 200 mL, urine output was 100 mL, and total fluid administration was 1,100 ml.

### Follow-up

**Figure 3:**
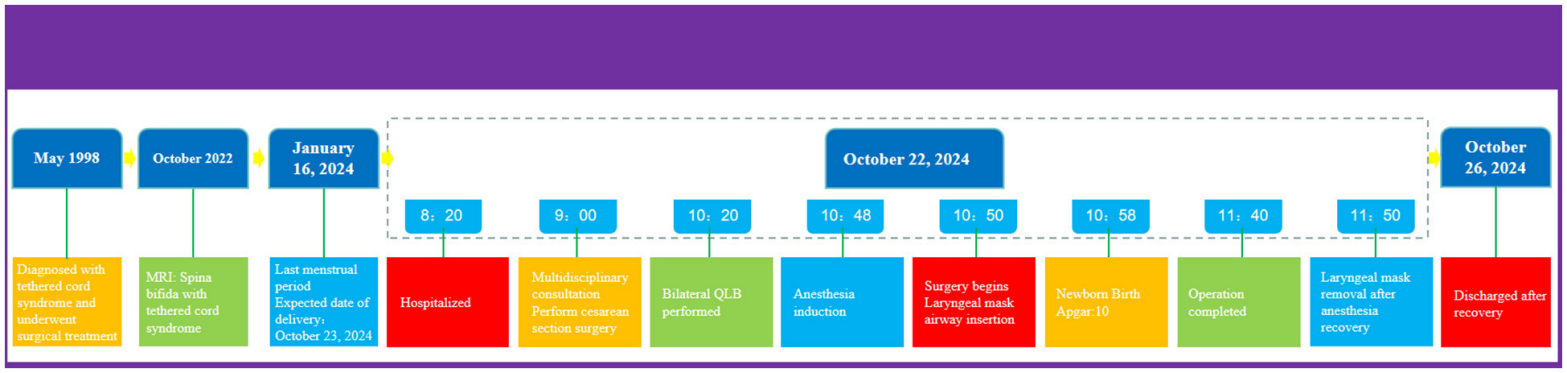
Treatment timeline of the patient.

The parturient had an uneventful postoperative recovery. Pain was well controlled, with visual analog scale (VAS) scores of 2 at 6 h, 1 at 12 h, and 1 at 24 h postoperatively. Parecoxib sodium 40 mg was administered intramuscularly every 12 h for supplemental analgesia. No complications related to GA were observed, and no adverse events associated with the nerve block (e.g., infection, hematoma, or motor impairment) occurred. The patient did not experience nausea or vomiting. The urinary catheter was removed at 24 h postoperatively, after which the patient was able to ambulate and resume oral intake. The surgical incision healed well without signs of infection. Neonatal follow-up at 72 h demonstrated stable vital signs, with no evidence of central nervous system depression (e.g., lethargy, poor responsiveness, or feeding difficulty). Neurobehavioral assessment, including level of consciousness, muscle tone, sucking reflex, Moro reflex, and alertness, was within normal limits. Both the mother and the neonate recovered well and were discharged on postoperative day 4. The timeline of the patient's treatment is shown in Figure 3.

## Discussion

GA is an important anesthetic modality for CD, particularly indicated in cases of maternal preference, emergency CD, contraindications to neuraxial anesthesia, or failed neuraxial block. In the present case, the patient had lumbar spinal deformity combined with tethered cord syndrome (confirmed by MRI) and declined neuraxial anesthesia; therefore, the indication for GA was clear. Compared with neuraxial techniques, conventional opioid-based GA carries potential risks such as neonatal respiratory depression, necessitating more rigorous preoperative preparation by the anesthesia team, including readiness for neonatal resuscitation and prevention of regurgitation and aspiration. While an opioid-free GA strategy with opioid administration deferred until after umbilical cord clamping confers neonatal benefits, one important potential limitation of this approach is the risk of insufficient intraoperative analgesia. In this case, bilateral QLB combined with opioid-sparing GA was employed to circumvent this limitation, using regional block as the primary analgesic component instead of systemic opioids, thereby effectively mitigating both neonatal and analgesic risks.

Regional nerve blocks provide effective analgesia. QLB can cover the dermatomal distribution from the thoracic intercostal nerves to the iliohypogastric and ilioinguinal nerves (13), with local anesthetic spreading closer to the paravertebral region. Compared with the transversus abdominis plane (TAP) block, QLB offers a broader range of sensory blockade, superior analgesic efficacy, and longer duration, and had been widely applied in abdominal surgeries, particularly in CD (9, 14). Currently, QLB is most commonly used for postoperative analgesia, while its application as a component of preoperative anesthesia remains relatively limited (15). According to the injection site, QLB can be classified into four types: QLB1 (lateral/classic), QLB2 (posterior), QLB3 (transmuscular), and QLB4 (intramuscular) (16). In this case, QLB1 was selected due to several advantages: it provides sufficient analgesia for surgical requirements, involves a relatively superficial injection site that reduces the risk associated with deep needle insertion, and offers a higher success rate given operator familiarity. Huang WK et al. verified that 0.5% ropivacaine yields rapid onset, sustained analgesia and robust early sensory blockade, significantly lowering postoperative VAS scores and cumulative opioid consumption. A total dose of 3 mg/kg is established as the safe, effective upper limit for single peripheral nerve blocks, delivering adequate perioperative pain control while minimizing the risk of local anesthetic systemic toxicity. Based on this safe total dose and the optimal 0.5% concentration (12), we selected an injection volume of 20 ml per side for the present case. Precise ultrasound-guided injection allowed clear visualization of fascial plane separation and medial spread of local anesthetic toward the spine, ensuring adequate coverage of the surgical incision.

Following confirmation of the sensory block coverage, rapid-sequence general anesthesia was initiated. Strict preoperative fasting guidelines were fully complied with throughout the perioperative period. Intraoperatively, continuous vital sign monitoring was implemented, alongside depth of anesthesia monitoring via Narcotrend and standardized neuromuscular transmission monitoring.

Anesthesia was induced with propofol combined with rocuronium. As a high-molecular-weight and highly hydrophilic muscle relaxant, rocuronium exhibits minimal placental transfer (2). Remifentanil was prepared merely as a rescue agent for insufficient intraoperative anesthetic depth, which was not administered due to stable intraoperative vital signs in this case. Supplemental opioids can be given after umbilical cord ligation once inadequate intraoperative anesthesia occurs, and this backup regimen is critical to ensure maternal and fetal safety. An opioid-sparing multimodal analgesic strategy was applied to control intraoperative nociception: preoperative intravenous parecoxib 40 mg was used for preemptive analgesia; bilateral quadratus lumborum block adequately relieved incision pain originating from the abdominal wall. Local anesthetic diffusion into the paravertebral space could partially alleviate visceral pain, whereas this analgesic effect was limited and highly individualized, failing to block visceral nociceptive transmission independently. Supplemental opioids are still optional after cord ligation when necessary. Meanwhile, obstetric surgeons adopted gentle and atraumatic surgical manipulation to reduce visceral traction stimulation. The combination of above analgesic measures contributed to opioid-sparing general anesthesia in this parturient.

Airway management was secured with a second-generation LMA Supreme. As validated in previous large-cohort studies, LMA application for cesarean delivery under general anesthesia does not elevate the risks of regurgitation, aspiration, or adverse maternal and neonatal complications when compared with conventional endotracheal intubation (17). Meanwhile, the LMA induces markedly less airway stimulation and hemodynamic perturbation, which further facilitates our opioid-sparing strategy. Notwithstanding the above benefits, full perioperative precautions against pulmonary aspiration were strictly enforced.

Anesthetic induction was deliberately timed closely prior to skin incision to shorten the fetal drug exposure interval. At the conclusion of surgery, residual neuromuscular blockade was fully reversed with sugammadex, enabling timely extubation and airway device removal. Dexamethasone was supplemented into the local anesthetic mixture to prolong the duration of regional analgesia, with postoperative VAS pain scores consistently maintained at ≤ 2 within the first 24 h. The neonate demonstrated no respiratory depression, achieved excellent Apgar scores at all time points, and required no resuscitative intervention. These outcomes further verify the safety and clinical feasibility of this multimodal anesthetic regimen in this high-risk parturient.

In summary, this approach combines the advantages of regional nerve block and GA, providing a feasible anesthetic strategy for parturients with contraindications to neuraxial anesthesia undergoing CD. Compared with conventional GA, bilateral QLB combined with opioid-sparing GA, offering superior incision-related pain control. However, QLB also has limitations: its efficacy for visceral pain was limited, it was not the sole analgesic modality and may be substituted by other regional techniques, and its application in emergency CD may be constrained by time requirements. In addition, when preoperative fasting was inadequate, the risk of aspiration with a laryngeal mask airway may increase; although endotracheal intubation offers greater airway protection, it was associated with stronger airway stimulation and may induce hemodynamic fluctuations, particularly in the absence of opioid supplementation. Opioids were reserved intraoperatively as rescue and supplemental analgesics to ensure anesthetic adequacy if required. This anesthetic approach was relatively novel, and clinical experience with bilateral QLB combined with opioid-sparing GA for cesarean delivery remains limited. Further studies are needed to validate its safety and efficacy.

However, the QLB also has important limitations. Its analgesic efficacy for visceral pain is limited, and it cannot serve as the sole analgesic modality; it may be substituted by other regional anesthetic techniques, and its application in emergency CD may be constrained by procedural time requirements.

With regard to airway management, important clinical trade-offs and considerations must be explicitly acknowledged. When preoperative fasting is inadequate, the risk of pulmonary aspiration is appropriately elevated with the use of a laryngeal mask airway. While endotracheal intubation remains the standard of care for airway management during CD and provides superior airway protection against aspiration, it is associated with more intense airway stimulation, which may induce significant hemodynamic fluctuations—particularly in the setting of an opioid-sparing anesthetic regimen, where no opioids are administered to attenuate this stress response. It was this specific balance of risks, combined with our patient's anticipated difficult airway and our overall opioid-sparing anesthetic goal, that informed our individualized clinical decision to use a second-generation laryngeal mask airway in this case. Opioids were reserved intraoperatively as rescue and supplemental analgesics to ensure anesthetic adequacy if clinically indicated. This anesthetic strategy remains relatively novel, and clinical experience with bilateral QLB combined with opioid-sparing general anesthesia for CD is still limited. Further large-scale prospective studies are warranted to validate the safety and efficacy of this approach in broader obstetric populations.

## Conclusion

We report a case of cesarean delivery successfully performed using bilateral QLB combined with opioid-sparing GA. In this approach, bilateral QLB was performed first, with confirmation of block efficacy and dermatomal coverage, followed by induction of GA and insertion of a laryngeal mask airway immediately prior to skin incision, without the use of opioids during induction. This strategy integrates the advantages of regional block and GA, potentially reducing the risk of opioid-induced neonatal respiratory depression; in this case, no respiratory depression was observed in the neonate. The mother achieved satisfactory postoperative analgesia without anesthesia-related complications. This approach may represent a safe and feasible alternative for parturients undergoing cesarean delivery who have contraindications to neuraxial anesthesia.

From the patient's perspective, the presence of spina bifida with tethered cord significantly increased the risks associated with neuraxial anesthesia. Although GA was considered as an alternative, concerns remained regarding neonatal respiratory depression, with opioids being a principal contributing factor. Despite counseling that strategies such as minimizing the induction-to-delivery interval and using ultra–short-acting opioids could reduce neonatal impact, GA is not the routine first-line technique for cesarean delivery, and perceived clinical experience remains relatively limited. Furthermore, as an advanced maternal age patient with a highly valued pregnancy, both the patient and her family were particularly risk-averse regarding neonatal outcomes. Following comprehensive multidisciplinary evaluation, the anesthetic team proposed a strategy of bilateral QLB combined with opioid-sparing GA, which was accepted by the patient. The anesthetic course was uneventful, with no neonatal respiratory complications, and the mother experienced smooth recovery and mild postoperative pain. The patient expressed appreciation for the multidisciplinary assessment and individualized management and was willing to share her experience to provide a reference for parturients in similar clinical situations.

## Data Availability

The datasets presented in this article are not readily available.
